# The effect evaluation of traditional vaginal surgery and transvaginal mesh surgery for severe pelvic organ prolapse: 5 years follow-up

**DOI:** 10.1515/med-2022-0467

**Published:** 2022-04-22

**Authors:** Ying-an Zhang, Wei Wang, Xiao-li Li, Xian-hui-Zhang, Jie Pan, Zhao-ai Li

**Affiliations:** Department of Gynecology and Obstetrics, Children’s Hospital of Shanxi and Women Health Center of Shanxi, Taiyuan, China; Department of Laboratory Medicine, Shanxi Provincial People’s Hospital, Taiyuan, China; Department of Pathology, Stanford University School of Medicine, Stanford, CA 94305, United States of America; Department of Gynecology and Obstetrics, Coal Hospital of Shanxi, Taiyuan, China

**Keywords:** pelvic organ prolapse, traditional vaginal surgery, transvaginal mesh surgery

## Abstract

The objective of this study was to compare the clinical effectiveness of traditional vaginal surgery and transvaginal mesh (TVM) surgery on severe pelvic organ prolapse (POP). We performed a retrospective chart review study of 258 severe POP patients who underwent surgery between November 2010 and September 2016. One hundred forty patients underwent traditional vaginal surgery and 118 TVM surgery. The Pelvic Organ Prolapse Quantitation (POP-Q) staging was used for objective evaluation. The Pelvic Floor Distress Inventory-20 (PFDI-20), Pelvic Floor Disease Life Impact Questionnaire Simplified Version-7 (PFIQ-7), and Pelvic Organ Prolapse/Incontinence Sexual Function Questionnaire (PISQ-12) were used for subjective evaluation. Their complications were also recorded. All the data were collected in the outpatient department through the follow-up at 3 months, 1, 3, and 5 years after the operation. Forty patients in the traditional vaginal surgery group and 25 in the TVM group were lost to follow-up. There was no difference in the POP-Q score between the groups (*P* = 0.346). The recurrence rate increased with follow-up time, reaching nearly 20% in the two groups by 5 years. The TVM group has higher PFDI-20 and PFIQ-7 scores and lower PISQ-12 scores than the traditional vaginal surgery group at six months, 1, 3, and 5 years, respectively (*P* < 0.001). Mesh exposure has occurred in the TVM group. Both surgeries showed similar objective satisfaction and recurrence rate. However, traditional vaginal surgery has higher subjective satisfaction than TVM in our study and does not risk exposure to prosthetic material.

## 1 Introduction

Pelvic organ prolapse (POP) is a common disorder with the female pelvic organs such as vagina, uterus, bladder, and/or rectum sagging downwards into or through the vagina, which seriously affects women’s physical and psychological wellbeing [1].

Recently, a nationwide population-based survey in China showed that the prevalence of symptomatic POP was 9.6% and increased with age [2]. To improve the quality of life, the lifetime risk of surgery in women with POP was 12.6–19% [3,4,5]. Transvaginal mesh (TVM) surgery, as minimally invasive surgery, has been widely used to treat POP in the past few decades. However, women who underwent the TVM surgery for POP have been reported to have increased complications and adverse events over time, including postoperative pain, mesh exposure, and discomfort of sexual intercourse [6,7].

TVM surgery has been questioned by people. In 2008 and 2011, the United States Food and Drug Administration (FDA) issued two public health notifications about the adverse events related to TVM. After that, the rate of TVM surgery has decreased significantly and been suspended in many countries [8]. However, some studies showed that the cure rate of mesh for POP outweigh conventional surgery [9]. Besides, some novel, ultralightweight meshes were also developed to treat POP to reduce the risk of adverse events [10]. Nevertheless, TVM surgery is still an alternative in China [11], and there are still controversies regarding which one is superior.

Traditional vaginal surgery is also called native tissue repair, which is non-mesh repair. Because of an increased risk of adverse events of TVM, interest in traditional vaginal surgery has re-emerged. Traditional vaginal surgery has been used for more than 100 years and has advantages, such as no foreign body implantation, fewer complications, and good outcomes. In the current study, to compare the clinical effectiveness of the two surgical techniques, we assessed subjective and objective outcomes in a large sample of patients with long-term follow-up.

## 2 Materials and methods

### 2.1 Study participants

All patients were recruited in the Women Health Center of Shanxi and the Coal Hospital of Shanxi between November 2010 and September 2016. This study was approved by the Ethics Committee of those two centers. Women diagnosed with Stage 3 POP or higher requiring surgical treatment were eligible for participation. The age of all patients ranged from 56 to 75 years old. Exclusion criteria: prior vaginal prolapse repair, pregnancy, abnormal liver and kidney function, coagulation dysfunction, endocrine diseases, and other medical diseases that compromise healing. All patients agreed to undergo the operation and signed written informed consent.


**Ethical approval:** This study was approved by the Ethics Committee of Women Health Center of Shanxi (2020020) and Coal Hospital of Shanxi (2020CH051).

### 2.2 Mesh and surgical technique

All surgeries are performed by the same gynecologist, a professional with more than 20 years of experience in POP surgery. Mesh materials used in our research are TiLoop total of 4 made in Germany.

Traditional vaginal surgery included an anterior median longitudinal colpotomy to reach the pubocervical fascia after performing a vaginal submucosal infiltration with diluted epinephrine solution. The anterior repair was performed by placing two layers of 2–0 synthetic absorbable sutures at the pubocervical fascia. The excess of the vaginal wall was removed and the vaginal mucosa was closed with continuous absorbable suture. A vertical incision in the posterior vaginal mucosa was made about the posterior. Then, the rectovaginal fascia was reconnected to the uterosacral ligaments at the top of the vagina, and sutured to the iliococcygeus fascia and muscle inferiorly to the ischial spines. Finally, reconstruction of the perineal body was performed. Vaginal skin closure was performed with delayed absorbable sutures.

In the implantation of TVM, the anterior arms were inserted through the fascia and muscular structures 1/3 of the upper obturator foramen (mainly the fascia of the external obturator muscle). The posterior arms were inserted through the arcus tendinous fasciae pelvis approximately 1 cm proximally from the ischial spine and brought out by obturator foramens onto the skin. About the posterior, mesh arms were inserted through the sacrospinous ligaments approximately 1 cm medially from the ischial spine, guided through the ischioanal fossa on both sides of the rectum and brought on the skin approximately 3 cm laterally and 3 cm below the external anal sphincter. Vaginal skin closure was performed with delayed absorbable sutures.

### 2.3 Evaluation indicators

**Figure 1:**
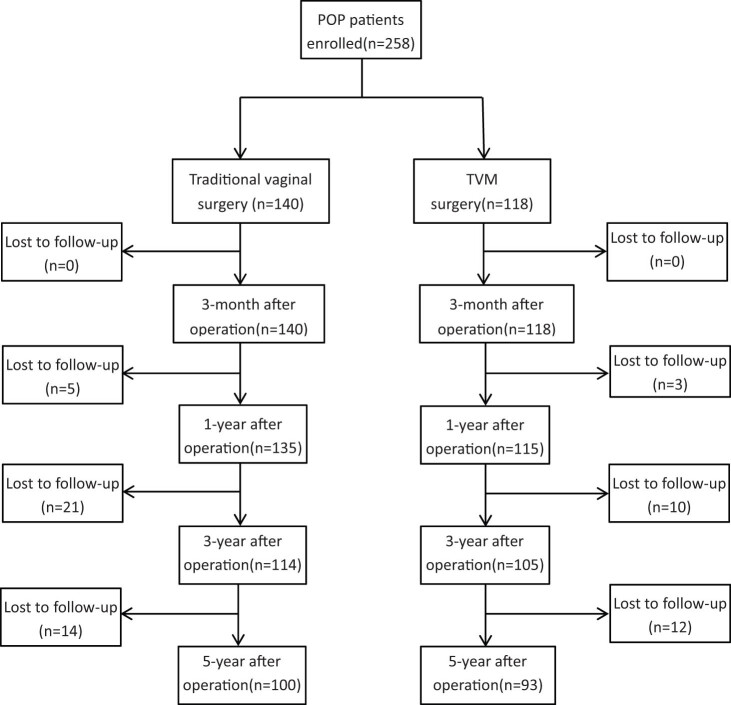
Flow chart of follow-up.

The study outcomes included the objective anatomic repair, function improvement, and complications. The accurate pelvic location restoration (the Aa, Ba, Ap, Bp, C, and TVL points) was evaluated by the Pelvic Organ Prolapse Quantification (POP-Q) score [12]. POP-Q score of grades I or below was considered as the cure. POP-Q stage ≥ II was defined as recurrence. The subjective evaluation was performed by three kinds of questionnaires, including the Pelvic Floor Distress Inventory-20 (PFDI-20) [13], Pelvic Floor Disease Life Impact Questionnaire Simplified Version-7 (PFIQ-7) [13], and Pelvic Organ Prolapse/Incontinence Sexual Function Questionnaire (PISQ-12) [14]. The PFDI-20 contains 20 items to assess symptoms of the pelvis, bowel, and bladder. Each item includes five options, from “asymptomatic” (0) to “extremely affected” (4). PFIQ-7 contains seven questions considering the bladder or urine, vagina or pelvis, bowel or rectal-related symptoms, and their social and mental health function. Responses to each question range from “not at all” (0) to “Quite a lot” (3). A high score means a greater impact on quality of life. PISQ-12 contains 12 items to evaluate sexual function. Each item also includes five options, from “not at all” (0) to “Quite a lot” (4).” Postoperative complications included vaginal foreign body sensation, pain, and mesh exposure. Two gynecologists performed the follow-up at 6 months and 1, 3, and 5 years after surgery. All the questionnaires and physical examinations were performed in each outpatient follow-up. The flow chart is shown in Figure 1. The loss to follow-up rate was 28.6% in the traditional vaginal surgery group and 21.2% in the TVM surgery group.

### 2.4 Statistical analysis

All data were analyzed by SPSS version 22.0 and GraphPad Prism 5 software. All continuous variables were presented using the mean ± SD or median and interquartile range. Independent samples *t*-test was used to compare continuous variables between the two groups. Recurrence rates are summarized by percentage and compared using chi-square or Fisher’s exact test. A *P* value <0.05 was considered statistically significant.

## 3 Results

### 3.1 Analysis of clinical characteristics, intraoperative and postoperative conditions

A total of 258 patients with POP were included. Which operation type to choose is based on the patient’s will. Of all patients, 118 underwent TVM surgery and 140 underwent traditional pelvic floor reconstruction surgery. There were no statistically significant differences in age, gravidity, and parity between the two groups (Table 1). All the patients underwent surgery successfully. There were no cases of vascular, bladder, or rectum injury.

**Table 1:** Comparison of patient clinical characteristics, intraoperative and postoperative conditions between the two groups

	TVM surgery (*n* = 118)	Traditional vaginal surgery (*n* = 140)	*P*
Age (year)	64.0 ± 2.0	61.0 ± 2.0	0.154
Gravidity	3.0 ± 1.0 (2–6)*	3.0 ± 1.0 (1–7)	0.579
Parity	2.0 ± 1.0 (1–6)	2.0 ± 1.0 (1–6)	0.956
Amount of bleeding (mL)	179.07 ± 61.78	176.82 ± 47.05	0.741
Urinary catheter removal time (days)	5.31 ± 1.02	5.24 ± 0.86	0.554
Hospital stays (days)	11.69 ± 1.87	11.78 ± 2.02	0.706
Duration of surgery (min)	185.6 ± 23.34	134.98 ± 17.37	<0.001

*The data in brackets are the minimum and maximum.

**Table 2:** Pre- and postoperative comparison of PFDI-20, PFIQ-7, and PISQ-12 scores between the two groups

		TVM surgery	Traditional vaginal surgery	*P*
Before operation	PFDI-20	104.70 ± 16.12	104.88 ± 15.82	0.928
	PFIQ-7	104.27 ± 19.12	101.66 ± 19.14	0.276
	PISQ-12	29.89 ± 6.95	28.71 ± 5.63	0.133
6 months after operation	PFDI-20	39.99 ± 6.52	31.40 ± 5.78	<0.001
	PFIQ-7	66.56 ± 12.35	54.36 ± 12.82	<0.001
	PISQ-12	—	—	—
1 year after operation	PFDI-20	30.93 ± 6.47	23.42 ± 4.17	0.001
	PFIQ-7	49.23 ± 11.01	39.42 ± 10.82	<0.001
	PISQ-12	50.68 ± 5.39	55.49 ± 5.78	<0.001
3 years after operation	PFDI-20	22.89 ± 4.13	16.38 ± 3.39	<0.001
	PFIQ-7	29.05 ± 3.06	25.06 ± 2.88	<0.001
	PISQ-12	69.63 ± 6.29	78.48 ± 7.72	<0.001
5 years after operation	PFDI-20	21.54 ± 4.32	15.73 ± 3.28	<0.001
	PFIQ-7	30.14 ± 3.37	25.00 ± 2.37	<0.001
	PISQ-12	69.44 ± 5.67	78.43 ± 7.36	<0.001

There was no significant difference in the blood loss volume, the length of hospitalization, and the indwelling catheter days between the two groups (Table 1). However, the operation duration in the traditional vaginal surgery group was significantly shorter than that in the TVM surgery group (*P* < 0.001).

### 3.2 Comparison of postoperative recurrent rate

**Figure 2:**
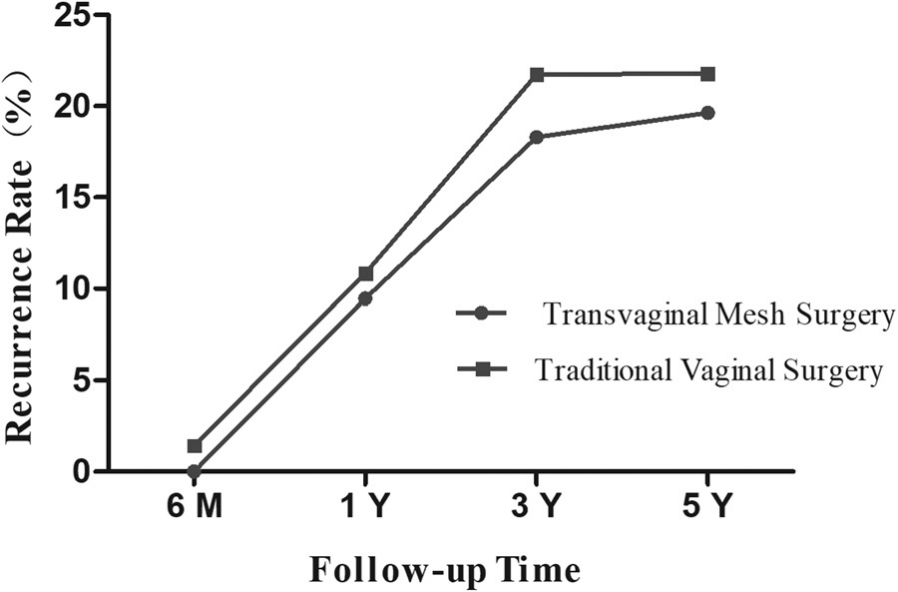
Comparison of the recurrence rate between the two groups with follow-up time.

We measured the POP-Q score of patients before and after treatment to assess the objective curative effect of two modes of operations. There was no significant difference in POP-Q score between the groups. The indicator points in the POP scoring system were all restored to the original anatomical position after the operation. POP-Q stage ≥II was considered as disease recurrence. At 6 months follow-up, there was no recurrence in the TVM surgery group and two cases of recurrence in the traditional vaginal surgery group. As shown in Figure 2, the recurrence rate increased significantly in both groups with follow-up time. At 5-year follow-up, the recurrence rate was 21.78% in the traditional vaginal surgery group and 19.64% in the TVM group. However, no statistical differences were found in the recurrence rate between the two groups.

### 3.3 Comparison of subjective symptom score

Before the operation, there was no significant difference in the subjective symptom score between the two groups (*P* > 0.05). After the operation, the PFDI-20 and PFIQ-7 scores in both groups were lower than before, and the PISQ-12 scores were higher than before the operation. At postoperative months 6, years 1, 3, and 5, all the subjective symptom scores of the traditional vaginal surgery group were better than the TVM group (Table 2).

### 3.4 Postoperative complications

In the TVM surgery group, two (1.69%) patients experienced vaginal foreign body sensations at 6 months after the operation, but no mesh exposure was found. The vaginal examination found that the anterior vaginal wall near the dome was thin, and the mesh could be touched under the mucosa. After being treated with estrogen ointment locally, one patient was improved with thickened vaginal wall mucosa 12 months after the operation. In contrast, the other patient still had mild vaginal foreign body sensation. Gynecological examination showed a local mesh exposure at the anterior vaginal dome, with a range of 0.5 cm × 0.5 cm. After trimming and applying external estrogen ointment for 2 weeks, the anterior vaginal wall recovered well and had no re-exposure. No postoperative complications were found in the traditional vaginal surgery group.

## 4 Discussion

Patients will undergo surgery with more than stage II POP or failed conservative treatment. Most POP surgeries are performed transvaginally and 10–20% via the transabdominal approach [15,16]. In China, TVM surgery and traditional vaginal surgery (non-mesh) are still two main transvaginal surgical methods in treating POP [17]. There remain some debates about the optimal surgical treatment for POP. Therefore, we performed this study to compare two operations’ cure rates, quality of life, and complications.

In China, even with the awareness of postoperative complications, the rates of TVM procedures account for nearly half. Sun et al. [17] analyzed the development of Chinese pelvic floor surgeries related to POP over the past 14 years. Their results showed that the rate of synthetic mesh operation increased from 38.1 to 46.0% and non-mesh procedures decreased from 61.9 to 54.0%. In our study, the TVM surgery group included 118 cases, accounting for 45.7% of all patients, consistent with the rest of the country.

Age, vaginal delivery, parity, and BMI are the most consistent risk factors for POP [18,19]. Therefore, to reduce the impact of these risk factors, our research included patients with similar age, gravidity, and parity in the two groups. In terms of perioperative parameters, there was no significant difference except the duration of surgery. The operation time of the TVM surgery group was longer. That is because we have to stop the bleeding carefully at every step of the TVM surgery operation to reduce the intraoperative blood loss resulting in mesh exposure. The patient’s overall health, mesh materials, and surgeon’s experience are also related to the POP operation [20,21].

The POP-Q was used to evaluate the objective effect of the two modes of operations. It is the most reliable POP grading system, which defines the anterior, posterior, and apical segment prolapse relative to the vaginal hymen and includes a five-point staging system [22]. POP was considered as recurrence when the POP-Q stage ≥ II. We analyzed the difference between the two groups with follow-up time. We found that the recurrence rate of the traditional vaginal surgery group is somewhat higher than the TVM surgery group, but this difference was not statistically significant. The results indicated that the two surgical methods were equally effective. In our study, the recurrence rate in both groups was approximately 20% at a 5-year follow-up. Ubertazzi et al. [23] also performed a 5-year follow-up outcome of TVM in 72 POP patients, and the cure rate was 79.2%, which is similar to our results.

The choice of surgical method is based not only on restoring typical anatomical structure but also on the functional recovery and patients’ quality of life, such as dysuria or abnormal defecation and dyspareunia. At present, there is little evidence about the subjective assessment of those two groups. Therefore, we analyzed the subjective symptoms by three questionnaires, including PFDI-20, PFIQ-7, and PISQ-12. After the operation, the subjective symptom scores were all distinctly improved in the two groups. Meanwhile, there were also statistically significant differences between the two groups. The results showed that the traditional procedure is better than TVM surgery in subjective effects. Six months after the operation, the PISQ-12 score has not been assessed as it is of no use.

Complications after TVM surgery were reported to have pain, mesh exposure, recurrent POP, sexual dysfunction, urogenital and rectovaginal fistulas, and so on [24]. Mesh exposure is a significant and common complication of TVM surgery. Previous studies reported the mesh exposure rate of TVM surgery was 3.1–16.6% [23,25]. However, in our study, only one case (0.87%) had mesh exposure 12 months after the operation. And, there are no postoperative complications in the traditional vaginal surgery group, which is much lower than other reports. The reason is probably that all patients before the operation had received conservative treatment, including antibiotics, topical estrogen, and 1:5,000 potassium permanganate sitz bath. And, during the process of puncture for mesh implantation, we emphasized that the implants must be placed in the interstitial space, not in tissue. Besides, all operations in our study were performed by the same experienced surgeon. For POP surgery, the doctor’s technique is also an essential factor. Slade et al. [26] have performed a systematic review including 27 randomized controlled trials to analyze the cost-effectiveness of surgical management of anterior POP. Their results showed that non-mesh repair has the highest probability of being cost-effective compared with all types of meshes. Our results also suggested that traditional vaginal surgery is the better procedure.

As a non-randomized controlled trial, our research has several limitations. First, the study has a small sample size. Second, the patients in our research are only from two centers. The multicenter study should be performed to verify the long-term prognosis. Third, we did not analyze conservative treatments like the pelvic floor muscle training after the operation. These data should be collected to make the study complete and more valuable. Besides, in recent years, the rate of laparoscopic sacrocolpopexy (LSC) for treating POP increased significantly because of the low incidence of complications and little effect on sexual function [27]. The laparoscopic lateral suspension with mesh was also considered as a safe and effective treatment for apical and anterior POP [28]. LSC should be compared with TVM and traditional vaginal surgery to provide more detailed information.

In our study, both surgical methods for POP are effective, but traditional vaginal surgery shows higher subjective symptom scores of postoperative satisfaction and no severe side effects than TVM surgery. Our result is similar to the final results of Boston Scientific Transvaginal Mesh for POP 522 studies completed on August 16, 2021 [29]. Their results showed that Boston Scientific transvaginal POP mesh had similar effectiveness and safety outcomes to native tissue repair at 36 months. However, the FDA believes that devices present potential risks and has ordered to stop selling and distributing products on April 16, 2019 [29]. Indeed, TVM surgery is still not prohibited in China. We hope our study will raise awareness of these types of surgeries. Besides, traditional vaginal surgery, due to high patient satisfaction, low rate of complications, low cost, and mature technology, is a valuable option for POP therapy.

## References

[1] Barber MD. Pelvic organ prolapse. BMJ. 2016;354:i3853. 10.1136/bmj.i3853 27439423

[2] Pang H, Zhang L, Han S, Li Z, Gong J, Liu Q, et al. A nationwide population-based survey on the prevalence and risk factors of symptomatic pelvic organ prolapse in adult women in China – a pelvic organ prolapse quantification system-based study. BJOG. 2021;128(8):1313–23. 10.1111/1471-0528.16675 PMC8252658 33619817

[3] Smith FJ, Holman CD, Moorin RE, Tsokos N. Lifetime risk of undergoing surgery for pelvic organ prolapse. Obstet Gynecol. 2010;116(5):1096–100. 10.1097/AOG.0b013e3181f73729 20966694

[4] Wu JM, Matthews CA, Conover MM, Pate V, Funk MJ. Lifetime risk of stress urinary incontinence or pelvic organ prolapse surgery. Obstet Gynecol. 2014;123(6):1201–6. 10.1097/AOG.0000000000000286 PMC4174312 24807341

[5] Wilkins MF, Wu JM. Lifetime risk of surgery for stress urinary incontinence or pelvic organ prolapse. Minerva Ginecol. 2017;69(2):171–7. 10.23736/S0026-4784.16.04011-9 28001022

[6] Ganj FA, Ibeanu OA, Bedestani A, Nolan TE, Chesson RR. Complications of transvaginal monofilament polypropylene mesh in pelvic organ prolapse repair. Int Urogynecol J. 2009;20(8):919–25. 10.1007/s00192-009-0879-9 19582383

[7] Morling JR, McAllister DA, Agur W, Fischbacher CM, Glazener CMA, Guerrero K, et al. Adverse events after first, single, mesh and non-mesh surgical procedures for stress urinary incontinence and pelvic organ prolapse in Scotland, 1997–2016: a population-based cohort study. Lancet (London, England). 2017;389(10069):629–40. 10.1016/S0140-6736(16)32572-7 28010993

[8] Zacche MM, Mukhopadhyay S, Giarenis I. Trends in prolapse surgery in England. Int Urogynecol J. 2018;29(11):1689–95. 10.1007/s00192-018-3731-2 30078099

[9] Ismail SI. Anterior colporrhaphy compared with collagen-coated transvaginal mesh for anterior vaginal wall prolapse: a randomised controlled trial. BJOG. 2014;121(11):1447–8. 10.1111/1471-0528.12830 25250934

[10] Deltetto F, Favilli A, Buzzaccarini G, Vitagliano A. Effectiveness and safety of posterior vaginal repair with single-incision, ultralightweight, monofilament propylene mesh: first evidence from a case series with short-term results. BioMed Res Int. 2021;2021:3204145. 10.1155/2021/3204145 PMC7801075 33490268

[11] Zhang L, Zhao Z, Chen J, Ma Y, Zhang G, Zhu L. Path-related pain after implantation of anterior transvaginal mesh: perspective from anatomical study. Int Urogynecol J. 2022. 10.1007/s00192-021-04924-6. 35034164

[12] Persu C, Chapple CR, Cauni V, Gutue S, Geavlete P. Pelvic organ prolapse quantification system (POP-Q) – a new era in pelvic prolapse staging. J Med Life. 2011;4(1):75–81. PMC3056425 21505577

[13] Barber MD, Walters MD, Bump RC. Short forms of two condition-specific quality-of-life questionnaires for women with pelvic floor disorders (PFDI-20 and PFIQ-7). Am J Obstet Gynecol. 2005;193(1):103–13. 10.1016/j.ajog.2004.12.025 16021067

[14] Zhu L, Yu S, Xu T, Yang X, Lu Y, Li B, et al. Chinese validation of the pelvic floor impact questionnaire short form. Menopause. 2011;18(9):1030–3. 10.1097/gme.0b013e31820fbcbe 21587092

[15] Clark AL, Gregory T, Smith VJ, Edwards R. Epidemiologic evaluation of reoperation for surgically treated pelvic organ prolapse and urinary incontinence. Am J Obstet Gynecol. 2003;189(5):1261–7. 10.1067/s0002-9378(03)00829-9 14634551

[16] Brown JS, Waetjen LE, Subak LL, Thom DH, Van den Eeden S, Vittinghoff E. Pelvic organ prolapse surgery in the United States, 1997. Am J Obstet Gynecol. 2002;186(4):712–6. 10.1067/mob.2002.121897 11967496

[17] Sun ZJ, Wang XQ, Lang JH, Xu T, Lu YX, Hua KQ, et al. A 14-year multi-institutional collaborative study of Chinese pelvic floor surgical procedures related to pelvic organ prolapse. Chinese medical journal. 2021;134(2):200–5. 10.1097/CM9.0000000000001237 PMC7817324 33443938

[18] Matthew DB. Pelvic organ prolapse. BMJ. 2016;354:i3853. 10.1136/bmj.i3853. 27439423

[19] Tineke FMV, Mirjam W, Joanna I, Kirsten BK. Risk factors for pelvic organ prolapse and its recurrence: a systematic review. Int Urogynecol J. 2015;26(11):1559–73. 10.1007/s00192-015-2695-8-73. PMC4611001 25966804

[20] Barski D, Otto T, Gerullis H. Systematic review and classification of complications after anterior, posterior, apical, and total vaginal mesh implantation for prolapse repair. Surg Technol Int. 2014;24:217–24. 24700225

[21] Nguyen JN, Jakus-Waldman SM, Walter AJ, White T, Menefee SA. Perioperative complications and reoperations after incontinence and prolapse surgeries using prosthetic implants. Obstet Gynecol. 2012;119(3):539–46. 10.1097/AOG.0b013e3182479283 22353951

[22] Bump RC, Mattiasson A, Bø K, Brubaker LP, DeLancey JO, Klarskov P, et al. The standardization of terminology of female pelvic organ prolapse and pelvic floor dysfunction. Am J Obstet Gynecol. 1996;175(1):10–7. 10.1016/s0002-9378(96)70243-0 8694033

[23] Ubertazzi EP, Soderini HFE, Saavedra Sanchez AJM, Fonseca Guzman C, Paván LI. Long-term outcomes of transvaginal mesh (TVM) In patients with pelvic organ prolapse: a 5-year follow-up. Eur J Obstet Gynecol Reprod Biol. 2018;225:90–4. 10.1016/j.ejogrb.2018.03.060 29680466

[24] Firoozi F, Ingber MS, Moore CK, Vasavada SP, Rackley RR, Goldman HB. Purely transvaginal/perineal management of complications from commercial prolapse kits using a new prostheses/grafts complication classification system. J Urol. 2012;187(5):1674–9. 10.1016/j.juro.2011.12.066 22425114

[25] Wei D, Wang P, Niu X, Zhao X. Comparison between laparoscopic uterus/sacrocolpopexy and total pelvic floor reconstruction with vaginal mesh for the treatment of pelvic organ prolapse. J Obstet Gynaecol Res. 2019;45(4):915–22. 10.1111/jog.13908 PMC6590650 30652385

[26] Slade E, Daly C, Mavranezouli I, Dias S, Kearney R, Hasler E, et al. Primary surgical management of anterior pelvic organ prolapse: a systematic review, network meta-analysis and cost-effectiveness analysis. Bjog. 2020;127(1):18–26. 10.1111/1471-0528.15959 31538709

[27] Serati M, Laganà AS, Casarin J, Gisone B, Cantaluppi S, Ghezzi F. Laparoscopic duplication of the uterosacral ligaments following hysterectomy for stage III-IV apical pelvic organ prolapse. Updates Surg. 2020;72(1):199–204. 10.1007/s13304-019-00690-9 31691118

[28] Mereu L, Tateo S, D’Alterio MN, Russo E, Giannini A, Mannella P, et al. Laparoscopic lateral suspension with mesh for apical and anterior pelvic organ prolapse: a prospective double center study. Eur J Obstet Gynecol Reprod Biol. 2020;244:16–20. 10.1016/j.ejogrb.2019.10.026 31770687

[29] https://www.fda.gov/medical-devices/urogynecologic-surgical-mesh-implants/fdas-activities-urogynecologic-surgical-mesh.

